# Is multi-drugs resistant Acinetobacter baumannii epidemic spread related to reduced susceptibility to biocides?

**DOI:** 10.1186/1753-6561-5-S6-P300

**Published:** 2011-06-29

**Authors:** B Casini, MG Minacori, A Buzzigoli, P Valentini, P Morici, S Barnini, C Tascini, F Menichetti, GM Rossolini, G Privitera

**Affiliations:** 1 Dep. Experimental Pathology, MBIE, University of Pisa, Pisa, Italy; 2 U.O. Igiene ed Epidemiologia, Azienda Ospedaliero-Universitaria Pisana, Pisa, Italy; 3 U.O. Microbiologia, Azienda Ospedaliero-Universitaria Pisana, Pisa, Italy; 4 U.O. Malattie Infettive, Azienda Ospedaliero-Universitaria Pisana, Pisa, Italy; 5 Dep. Molecular Biology, University of Siena, Siena, Italy

## Introduction / objectives

Aim of this study was to assess the role of active extrusion in mediating decreased susceptibility to biocides in the persistance and spread of multi-drugs resistant of *A. baumannii* strains isolated in intensive care units of a tertiary-care teaching hospital.

## Methods

67 clinical and 24 environmental strains isolated from 2007 to 2010 were genotyped by PFGE, MLST and REP-PCR. Multiplex PCRs were performed for identification of the *ompA*, *csuE* and *bla*_OXA-51like_ sequence type groups. The antimicrobial susceptibility was determined and the presence of carbapenemase-encoding genes was analysed by characterization of the bla_OXA_ genes. Chlorine susceptibility was analysed according to BS EN 1040:1997

## Results

The cross-analysis of genotyping methods allowed to group strains into 4 clones, only the clone A belonging to the Group 1 corrisponding to European II clonal complex and the clone B belonging to Group 2 clonal complex European I. Since 2008, a new variant of clone A has emerged as the predominant clinical and environmental strain, resulting positive for the presence of the carbapenemase OXA-58 plasmid-mediated and showing reduced chlorine susceptibility *in vitro*

## Conclusion

Multi-antibiotic resistance may not be the only strategy applied by *A. baumannii* to spread and persist in healthcare setting, but also the increased resistance to biocides

## Disclosure of interest

None declared.

